# Schrödinger's Cat—Parallel experiences: exploring the underlying mechanisms of undergraduates' engagement and perception in online learning

**DOI:** 10.3389/fpsyg.2024.1354641

**Published:** 2024-07-16

**Authors:** Hongfeng Zhang, Yan Liu, Shaodan Su

**Affiliations:** ^1^Faculty of Humanities and Social Sciences, Macao Polytechnic University, Macau, Macao SAR, China; ^2^Faculty of Education and Human Development, Department of Curriculum and Instruction, Hong Kong, Hong Kong SAR, China; ^3^Foshan University, Foshan, Guangdong, China

**Keywords:** online learning perceptions, learning motivations, learning engagements, self-determination theory, parallel experiences

## Abstract

**Background:**

The emergence of e-learning had an intense, immediate, and disruptive transformation in the education system. While education aims to seek more interactions and learning engagement between teachers and students, it turns out that it takes lots of work to achieve the goal in the online classroom.

**Aims:**

This study aims to explore the underlying mechanisms and implications that emerge from the influence of the new features of online learning, drawing on students' real-life experiences, to construct a comprehensive theoretical model.

**Sample:**

From July 2023 to October 2023, 56 undergraduates, including 18 male and 38 female, participated in the data collection process either face-to-face or online.

**Methods:**

The study constructs a substantive theoretical model by employing the approaches of The Grounded Theory, three level-coding constant comparative method, theoretical sampling, core category distillation and storyline collation.

**Results:**

(1) The elements involved in the process of online learning exhibit underlying logical correlations, driven by profound underlying factors, ultimately resulting in a parallel experience akin to Schrödinger's Cat. (2) Online features lead to students' motivation mainly depending on whether they are self-regulated. (3) Teacher-student interactions and self-regulation shape different learning contexts and types by being moderated by internal and external effects.

**Conclusions:**

Students had a parallel experience similar to that of Schrödinger's Cat: they were constantly dissatisfied with “rational” learning and “perceptually” satisfied with online learning. The variation in the satisfaction of the three basic psychological needs necessities college students in online learning leads to parallel experiences.

## 1 Introduction

The advent of e-learning marked a profound, immediate, and transformative shift in the education system (Garrison et al., 1999). Despite the numerous advantages that e-learning offers over traditional classrooms, a lingering question persists: has e-learning truly delivered on its promises? (Anderson, 2008, p. 91). Debates surrounding our educational objectives are intricately tied to disagreements about the merits of e-learning technology (Kanuka and Kelland, 2008). The use of technology in education has both positive and negative aspects. Before the pandemic, traditional education systems showed little interest in online learning, as reflected in limited academic discussions and minimal integration into classrooms. Online learning will be more sustainable while instructional activities will become more hybrid provided the challenges experienced during this pandemic are well explored and transformed to opportunities (Adedoyin and Soykan, 2023). In this era, the technologically advanced online education system continues to capitalize on its unique strengths.

While online education is not a new concept, students lack extensive experience with it. The shift to online learning has brought forth various challenges, such as communication gaps between teachers and students (Vlachopoulos and Makri, 2019), decreased student engagement (Yang et al., 2018; Purarjomandlangrudi and Chen, 2020), alienations of assessment functions (Conrad and Openo, 2018; Zhang et al., 2021) and reductions in student's learning motivation and autonomy (Luburić et al., 2021). Educators are grappling with unexpected difficulties due to the technical changes imposed by online education, and these challenges persist. The validity of students' motivation and engagement is a crucial concern, and these issues in online learning are interconnected. For instance, an “eLearning Contradiction” exists between the desire to integrate technology with learning and the reality in most classrooms (MacDonald et al., 2005). This is influenced by varying student perceptions and the level of alignment between online learning and educational goals. This study, set against the backdrop of the shift from offline to online learning, aims to explore the underlying mechanisms and implications of the new features of online learning. Drawing on students' real-life experiences, the goal is to construct a substantive theoretical model. Grounded theory, known for exploring natural features and constructing substantive theories, is employed due to its strength in qualitative methodologies. This approach, utilizing constant comparison and data coding analysis, aligns with the research aims, as quantitative research on causality is limited in developing a theoretical framework (Graebner et al., 2023).

## 2 Literature review

### 2.1 Online learning perceptions

Online learning, utilizing the Internet for access to materials and knowledge, involves students interacting with teachers and peers to enhance their learning experiences (Ally, 2004). Amid the epidemic, research has predominantly focused on students' perceptions of online learning amid contextual shifts. Studies have explored holistic aspects such as social presence, interaction, and satisfaction in comparison to face-to-face learning (Bali and Liu, 2018; Conrad et al., 2022). Additionally, research has delved into perceived differences in knowledge and skill acquisition between students and teachers (Syauqi et al., 2020), encountered barriers in psychology, technicity, and academia during online learning (Gonzalez-Ramirez et al., 2021), and varied experiences of students across different dimensions and countries (Cranfield et al., 2021).

Another set of research delved into more specific aspects. Some papers focused on factors affecting students' online learning performance, revealing that computer/Internet self-efficacy directly impacted course satisfaction (Wei and Chou, 2020). Martin and Bolliger (2018) explored students' perception of engagement strategies in online courses, finding that learner-to-instructor engagement strategies were highly valued. Previous research investigated undergraduates' experiences with and preferences for popular online learning applications during COVID-19 (Hendrawaty et al., 2021). Studies also examined how students' trust in their capacity to learn online, self-discipline plans, and confidence in using online learning platforms could predict their satisfaction and perceived usefulness of e-learning (Landrum, 2020). Addressing cultural diversity, Kumi-Yeboah et al. (2022) focused on minority students' online learning perceptions, highlighting the significance of diverse cultural experiences in online learning environments. Despite these diverse perspectives, existing research often dissects students' online learning perceptions into independent parts without establishing correlations and logical associations. Hence, there is a valuable opportunity for in-depth exploration in this area.

### 2.2 Online learning motivations

Online learning motivations significantly influence students' behaviors and outcomes, with studies highlighting their importance (Hartnett, 2016). Factors influencing and impacting engagement (Kim and Frick, 2011; Abdous, 2019; Esra and Sevilen, 2021) and achievement in online learning motivations have been explored (Lim and Kim, 2003; Dunn and Kennedy, 2019). Common causes include interest in the topic, sharing expertise, and relationship development, demonstrating complementary motivations among participants with different areas and levels of expertise (Gilbert, 2018). Understanding the mechanisms behind learning motivation is crucial, as these variables play a significant role in predicting students' learning success (Credé and Phillips, 2011). Research indicates lower motivation levels among online students compared to face-to-face students (Stark, 2019). Addressing this, some studies advocate for incorporating game dynamics to support natural learning motivation (Kam and Umar, 2018). Examining the association between students' game-playing motivations and gamification elements in higher education, researchers suggest tailoring gamification efforts to engage and motivate students of different levels (Jaskari and Syrjälä, 2022). The widely implemented self-determination theory has been applied to investigate the influence mechanisms and impacts of online learning's preparation level and learning engagement (Bovermann et al., 2018; Chiu, 2022). Students with a relatively low level of preparation exhibit non-autonomous motivation (Bovermann et al., 2018). However, further exploration is needed to understand how the self-determination theory operates in students' more profound online learning experiences.

### 2.3 Online learning interaction and engagement

When large-scale online learning is launched, more attention is paid to how interaction is carried out under the human-computer-human learning format and the impact of motivation and interaction on online learning participation and outcomes. For the interactions, some scholars explored the many types of connection and the multidimensional characteristics of communication in online learning settings (Vlachopoulos and Makri, 2019); how was interpersonal interaction measured (Mehall, 2020); the associations between online interactions and engagement (Purarjomandlangrudi and Chen, 2020). Interactions are a necessary part of online learning. Its primary purpose is to enhance students' learning effectiveness.

Therefore, students' perception of engagement strategies implemented in e-learning based on the interaction framework is critical (Martin and Bolliger, 2018). Domina et al. (2021) thought that learners with advanced technological skills interact more deeply. Some papers assessing online learning engagement suggested that perceived learning satisfaction predicts learning engagement in online learning courses (Chan et al., 2021). Besides, the online assessment conducted among students is tightly connected with online techniques and environment (Spitzer et al., 2021). Based on various forms of engagement, a study showed the outcomes of real-time online learning. It was shown that student academic outcomes are more significantly impacted by cognitive and behavioral involvement (Kobicheva, 2022).

From the former studies, online learning perception is an issue resulting from the swift learning methods. The subjects under discussion are no matter the perceptions' consequences, online learning's start (motivation), process (interaction and engagement), and outcomes. There are many significances in prior research. Nevertheless, the components of online learning are rarely viewed as a continuous, interacting, and logically related whole. This study intends to take a theoretical approach based on The Grounded Theory to the various aspects of students' online learning to connect them into a logical “storyline”. By doing so, insights into the underlying connotations and deeper reasons behind the perceptions of widespread online learning will be helpful to construct a corresponding theoretical framework.

## 3 Methodology

### 3.1 Sampling and data collection

This study's sample selection was mainly based on undergraduates from universities in Macau, supplemented by choosing students from universities in neighboring areas as auxiliary and external validity test samples. Macau is a small region with ten universities, four public and six privates. The main reasons for choosing students in Macau as the research participants are as follows. First, Macau is a multicultural region. College students have cultural characteristics from different countries and areas. The teaching language is mainly English. Learners from various cultures absorb their cultural practices in different ways, and there are cultural differences in their perceptions and how their basic needs are met (Ryan and Deci, 2020). Therefore, it is possible to conduct the study with mainly Macau university students, which is more representative and generalizable. Second, Macau universities admit a more significant proportion of mainland Chinese students. The admission criteria of these students are the first batch of admission marks in the Chinese college entrance examination, with higher academic standards, representing a higher quality group among universities. Third, more than half of local high school students in Macau go abroad for further studies. Those who stay in local universities have weaker academic standards and cultural backgrounds significantly different from those of mainland students (Su et al., 2022). Fourth, according to the government's plan, a certain percentage of international students are enrolled in Macau universities. This group of students also mainly receives subject education, which is typical of online classroom learning interaction and perception.

From July 2023 to October 2023, 56 undergraduates (18 male and 38 female) participated in the data collection process either face-to-face or online. Sampling methods included typical sampling, theoretical sampling, random sampling, and others. In July 2023, the sampling was mainly focused on one public university P in Macao, and the researchers sampled a total of 15 college students in the fields of Humanities and Languages, Public Administration and Social Sciences, and Medicine and Exercise Physiology, with typical and random sampling (see below for the basis and method of sampling); and in September, the sampling was continued in college P, another public university M, a private university C and neighboring areas based on the constant sampling principle. A total of 24 college students were added to the sampling, including those majoring in Tourism, Artificial Intelligence, and Education in Social Sciences. After the second round of sampling was completed, based on the results of coding and sorting, the researcher still felt the lack of many properties and dimensions under the deductive logic in the process of writing the article, and the research team decided to conduct focus interviews in three times in October 2023 through a combination of random sampling and theoretical sampling by sampling a total of 17 college students of the above-mentioned majors in University P again.

The research team developed a semi-structured interview outline in advance, which included demographic information; characteristics of online classes (such as time, space, content, activities, and materials); adaptability of online learning (process and assessment); online learning technologies (software, facilities, and their impacts); interactivity of online learning; engagement and effectiveness of online learning (including cognition, emotions, skills, behaviors and so on); and improvements in online learning. Specific interview questions were based on this framework. Follow-up questions will be posed to the interviewees, particularly when significant discourses or metaphors that contribute to research questions and theory development are identified. These insights will serve as the foundation for subsequent theoretical sampling. After obtaining ethical permission and verbal consent from the students interviewed, the in-depth interviews were recorded and transcribed into transcripts. The interviewees were coded in a four-level format, for example (M, F)–(SS, H, S)–(M, O)–230920. Represents (Male, Female)–(Social Science Major, Humanities Major, Science Major)–(Macau, Outside Macau)–Interview date 20th, September 2023.

The study drew primarily on The Grounded Theory introduced by Strauss and Corbin (1990) and Corbin and Strauss (2014). In keeping with the principle of theory formation based on empirical data, we used the constant comparative method to explore the “core category” of college students' perceptions of online learning (Hallberg, 2006). The data collection, analysis, and theoretical model generation were carried out alternately. In the process of the study, we maintained a constant comparison with the theory and literature based on the logical sequence formed by the data analysis to ensure the reliability and validity of the research findings.

### 3.2 Constant comparisons and data analysis

In the initial sampling stage, a targeted approach involved selecting two local Macau students, one Mainland student, and one international student for in-depth interviews. Transcripts were then analyzed using the first-level coding procedures of Grounded Theory (Strauss and Corbin, 1990). Researchers conceptualized interview scripts, assigning conceptual names based on generalizations or intuitive phenomenological presentations. Preliminary categories were formed, uncovering properties and dimensions, and integrating the three. Utilizing cultural characteristics in representative sampling enhances a comprehensive understanding of online learners' characteristics, streamlining subsequent sampling stages.

The researchers will employ a logical deductive-based theoretical sampling approach alongside random sampling for comprehensive comparison and analysis. Theoretical sampling, following a deductive model, involves categories, properties, and dimensions identified through representative sampling that are not saturated. Based on findings and deductions, researchers will establish the theoretical target sampling. For instance, logical deduction of “patterns” suggested conceptual dimensions related to “bidirectional,” leading to the adoption of a theoretical sampling approach to explore specific “bidirectional” patterns, such as the “asynchronous” pattern in online learning. This pattern, identified during representative sampling, involves deeper investigation, and students with limited interactive connections will be purposely selected for interviews. This contributes significantly to refining concepts within theoretical sampling. The research team also conducted random sampling within a sample frame, organizing three focus group interviews to fill gaps in theoretical sampling, test reliability and validity, and assess theoretical saturation robustness through interactive interviews.

After three rounds of data collection and coding analysis, the study formed complete categories, properties, and dimensions. Please see Table 1.

**Table 1:** Categories, properties, and dimensions.

**Categories**	**Properties**	**Dimensions**
Spatiotemporal flexibility	Spatial flexibility	Home, anywhere
	Temporal flexibility	Anytime, repeat
Human-Computer-Human	Pattern	Unidirectional, asynchronous
	Perception	Advantages, disadvantages
Learning changes	Self-regulatory status	Self-discipline, mind wandering
	Interconnection	Strong, weak
Engagement differentiation	Logical engagement	Seldom communication, low efficiency
	Emotional engagement	Relaxed, stress-free
External influences	Internet technology	Unstable, unfamiliar
	Learning environment	Spatiotemporal combination, alienation of assessment
Pedagogy strategies	Teaching methods	Down to earth, dull
	Teaching content	Theories, practices
Parallel experiences	Logical dissatisfaction	Low learning marginal effect, not fully understand
	Emotional satisfaction	Freedom, autonomy

### 3.3 The core category and storyline association

After identifying categories, properties, and dimensions, this study follows The Grounded Theory's storyline organization for core category refinement.

Online learning during the epidemic, a forced adoption, exhibits advantages and disadvantages in college studies due to human-machine-human interaction and spatiotemporal flexibility. Students' motivation depends on self-regulated capacity, influenced by teacher-student interactions, guided by instructors, and the level of self-regulation. External factors (technology and environment) and internal factors (teaching strategies and content) moderate this process. For instance, gamification teaching can engage less self-disciplined students, leading to logical learning satisfaction. Regardless of the engagement mode, students have parallel experiences akin to Schrödinger's Cat, being “logically” dissatisfied but “emotionally” satisfied with e-learning. In summary, online learning's ultimate experiences are shaped by essential psychological needs and rules.

From this, we distill the core categories of this study. The storyline begins with the characteristics of online learning and concludes with the profound experiences it generates. Whether it's students' motivation and engagement levels, or the impact of internal and external interventions, these remain merely “contingent” factors in online learning. The “inevitability” determinants of online learning status lie in internal psychological needs and understanding patterns. Hence, the study identifies “Schrödinger's cat-parallel experiences” as the central category around which all “contingent” and “inevitable” factors revolve.

**Figure 1:**
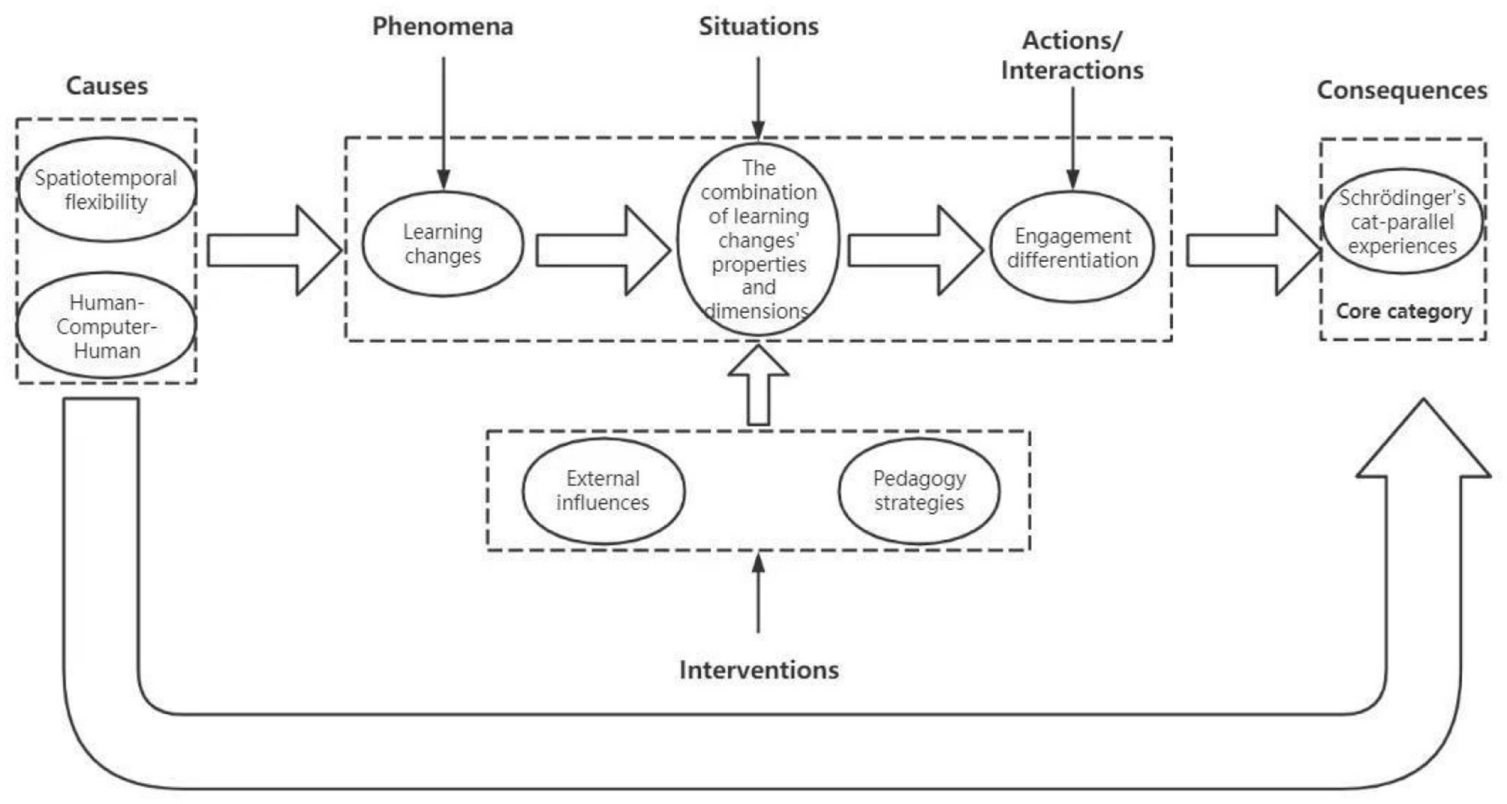
Undergraduates' online learning code format model.

The coding paradigm model of this storyline is shown in Figure 1.

## 4 Model interpretation

### 4.1 Causal condition: Human-Computer-Human and spatiotemporal flexibility

Research shows that the shift from offline to online significantly changes college students' learning. This is mainly determined by online learning's Human-Computer-Human and Spatiotemporal flexibility characteristics. The so-called online where a screen separates teachers and students, is the opposite of offline, face-to-face teaching. In this learning model, teachers and students can freely talk to each other. However, in the actual situation, the teacher or the students often only output in a “one-direction” way. *We rarely turn on the camera. Moreover, when we answer questions, we feel like just talking to ourselves with no one physically in front of us*. (F-H-M-230916) Even if there is a two-way interaction between teachers and students, it is mostly lagging and belongs to the “asynchronous” mode. *After all, teachers and students are separated, and even in the discussion forum, statements can only be seen after refreshing (the website)*. (M-SS-O-230918) The freedom to learn at your own pace, the option to stay at home, and the comfortable settings were the most often cited benefits of the Human-Computer-Human mode (Baczek et al., 2021). *However, the biggest problem is you cannot see your classmates and do not feel like going to school anymore*. (M-S-O-230711) Besides, the online learning atmosphere could be more exciting and take time to adapt. Facing the computer for a long time makes many students feel physically fatigued, decreasing their learning efficiency.

However, the spatiotemporal flexibility of online learning also brings another kind of perception to most students. On the one hand, there is no restriction on location for learning. While most students take classes online at home, others take classes in libraries, cafes, and dormitories. There are no rigid restrictions compared to face-to-face instruction. On the other hand, time is flexible. *I can look for information about the teacher's lecture on my computer at any time…The teacher's PowerPoint or recorded video can be accessed again and again. This feeling is different from offline*. (F-H-O-231012)

Accordingly, the advantages and disadvantages of online learning constantly interact together and even transform each other. For example, *when I was in the dormitory by myself, it was free indeed. However, with computers and cell phones around, it is easy to get distracted during online classes. Unconsciously, I play games or fall asleep*. (F-SS-M-230713) The reason for this phenomenon is closely related to the changes that have taken place in learning, which are concentrated in the areas of motivation and interactive connections. This phenomenon occurs because of learning changes focused on motivation and interactive connections.

### 4.2 learning changes: a contextual field of motivation and interaction

**Figure 2:**
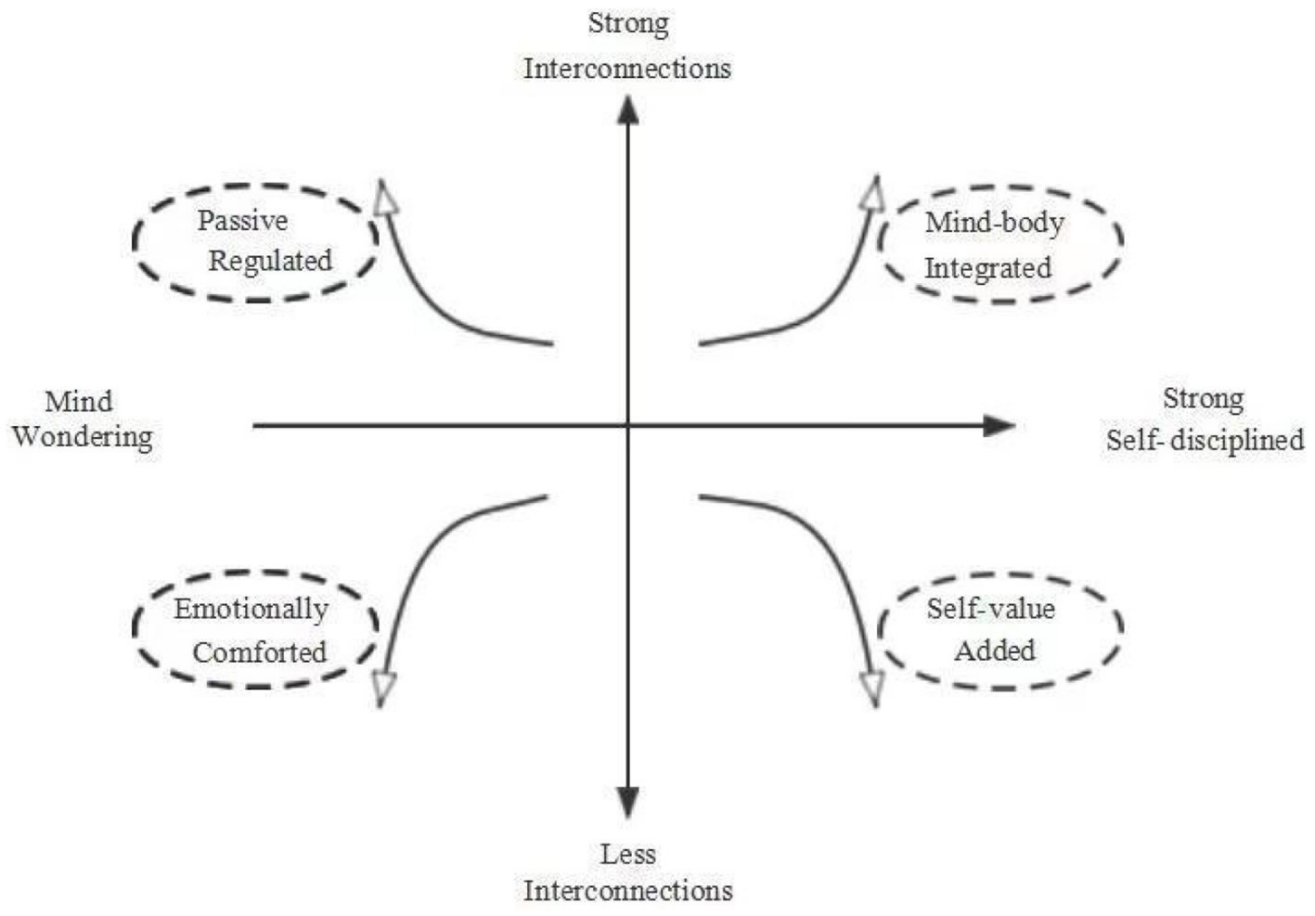
Properties and dimensions combination quadrant diagram for online learning change.

“Offline” and “online” are seemingly analogous terms; however, from the student's viewpoint, the characteristics of Human-Computer-Human interaction and spatiotemporal flexibility engender significant shifts in the learning paradigm. The category of learning change encompasses two attributes: “motivation” and “interconnection.” The contextual domain is shaped by the amalgamation of various dimensions of these two properties, giving rise to four distinct types of online learning changes for students: Mind-body integrated, Passive regulated, Emotionally comforted, and Self-value added. Please see Figure 2.

Quadrant I combines “strong” interconnection and self-regulatory status “self-discipline”. Students who move to the upper right of this quadrant tend to be more Mind-body integrated. This type of student has clear study goals and a detailed life plan. *I need to make a detailed plan for myself every week and organize tasks at each stage*. (F-S-O-230916) They not only deeply know the meaning of learning but also can experience the joy of learning. *If teachers and students are active enough in their interactive connections, including active discussions, deep thinking, and high levels of participation, it can lead to complete immersion in learning*. (F-H-O-230711) Motivation is a topic that many scholars have discussed. Some students enter the classroom with a “motivation to think”. No matter what the teacher does, these students always adjust their mindset to learning. These students fully engage and reflect on their thoughts and actions when the classroom is vivid and exciting. *When teachers teach online with more interactions, you will be so eager to share that you will type on the computer and send it up immediately. In offline learning, you will not have this urge. After all, you are too embarrassed to interrupt the teacher's class*. (M-SS-O-231012)

Quadrant II combines “strong” interconnection and self-regulatory status “Mind wandering”. Students toward the upper left of this quadrant tend to be passive regulators. In the interviews, a large percentage of the students believed they had low self-discipline. Learning can be affected by mind wandering and attention distracted from a task, especially in increasing online learning environments (Randall et al., 2022). *Your brain automatically identifies the home as a place to rest when you take online classes at home, making it difficult to focus on and control your learning*. (F-H-M-230920) So, motivating students to learn is much harder if teachers do not shift their offline teaching mode. *The teacher* ^***^has the common touch, teaching knowledge just like telling the story. Also, he constantly sends emoticons and small red flowers. The interaction in the comment section of this teacher's class has been very active since before the class started. (F-H-M-230715) The learning environment partly determines motivation for online learning. In this kind of environment, students' expectations have been lowered. Even when teachers have a strong theoretical capacity and coherent teaching, some students still easily forget the knowledge. However, when teachers can better interact with students and add more practical sessions, students, although in a passive environment, will be positively conditioned by the change in the teacher's interactive approach and practical feedback. *I would be touched by the teacher's relaxed yet colorful approach. Even after class, I will reflect and consider how some questions should have been better answered*. (M-SS-O-230920)

Quadrant III combines “weak” interconnection and self-regulatory status “Mind wandering”. Students toward the lower left of this quadrant are emotionally comfortable. Traditionally, students are believed to become freeride once these two are combined. However, online education is unique because this group of students is more accustomed to a relaxed, comfortable, and free atmosphere. *I did not feel uncomfortable (learning online). Moreover, I was more relaxed than in in-person classes, in which I felt very pressured, especially when answering questions because I felt like I was in the spotlight*. (F-SS-M-230713) Other students may think that online learning is more advantageous because they are easily distracted by other things in face-to-face classes. *Physically, you have to care about wearing, makeup, arrival time, and even who is sitting around you. Unlike online, you do not have to think about these things at all*. (F-SS-O-231012) Even those students who think they have weak self-control want to take advantage of the online time and space flexibility to initiate change themselves. *Online classes make me lax, and the professor does not try to create an interactive atmosphere. In order to keep myself from falling too far behind, I need to go online to find some information myself.....*. (F-SS-M-230918) For this kind of student, the biggest advantage of online classes is that they feel comfortable and do not have the tension they feel when studying offline. This experience can even replace the real purpose of learning.

Quadrant IV combines “weak” interconnection and self-regulatory status “self-discipline”. Students in the lower right of this quadrant tend to be self-value-added. Due to the nature of the online classroom, the interaction could be better. *When teachers find that interaction is not very effective, I find that they tend to lecture more*. (M-S-O-230718) Self-disciplined students can turn online disadvantages into advantages. The teacher does not clarify it, and constant listening can be exhausting. *I would go online to find relevant information on my own and combine it with what seemed uninteresting in the lecture. I would also discover knowledge that I had not known before, which was an exciting process (with an expectant look).......*. (M-SS-O-231018) Suppose such a process of knowledge growth is “unintentional” during boring learning. In that case, another group of students has an active self-regulatory strategy and can learn at their own pace. It has been proved that using self-control techniques significantly enhances motivated learning behavior (Pawlak, 2022). Furthermore, self-regulatory structures are thought to be the main elements influencing long-term engagement and academic performance (Sun et al., 2018). *I do not require myself to pay full attention in every class. I mainly like online classes because of the opportunity to access class records and playback. When I study via video and playback, I can study at my own pace. I can pause, take notes, and look up information anytime. So I do not care how the teacher teaches, because I study again after the class*. (F-H-O-230920) Students with strong self-discipline need the possibility to acquire knowledge and only care if the platform for acquiring knowledge is provided. With strong interactions, students with strong self-discipline are more fully engaged; with weak interactions, students with strong self-discipline look for paths to self-improvement.

### 4.3 Engagement differentiation: emotional and rational engagement with internal and external interventions

In the face of changes in the learning transition process and challenges in students' adaptation levels, students differentiate between rational and emotional engagement. Changes in the level of engagement in response to internal and external conditions manifest this differentiation.

#### 4.3.1 Logical engagement: low communication and low efficiency

No matter which type the students belong to, compared to their more conventional classroom colleagues, students were less likely to engage in student-faculty exchanges and dialogue with people from different backgrounds (Sun et al., 2018). *Lack of communication makes me feel difficult to understand new knowledge. When learning online, I cannot see others physically. Then I will be free and lazy, decreasing my learning efficiency*. (M-S-O-230711) Even students with a high level of self-discipline are the same, but objective circumstances can sometimes be changed according to personal requirements.

*I can easily be inattentive in online classes. Some teachers send out expressions, use Internet buzzwords, self-comparison as an internet influencer, gamification and other grounded ways. It is easy to remember that knowledge point. I need help understanding where I will take the initiative to look up after class*. (F-H-M-230715)

*In online learning, I am less focused than offline! Nevertheless, I know I will definitely record and listen back as I can. I do not care if the online efficiency is lower because I am taking listening back as my real learning*. (F-H-O-230920)

Normally, the student's perceptions of communication and efficiency are connected. The internal and external intervention conditions (external influences and pedagogy strategies) were activated as moderating variables in their engagement actions. The “external influences” included “ Internet technology” and “learning environment”. Most students felt distributed by technological instabilities such as network lags and dropped connections. Sometimes, it affects learning because *communication is already poor, and when the network is stuck, you cannot hear anything*. (F-SS-M-231018) Sometimes, it also affects the teacher-student relationship because *when a student is asked a question, he cannot answer because of the poor connection. The teacher may mistakenly think that the student is not there, or he does not want to answer, causing misunderstanding*. (F-SS-M-231012) In addition, some older teachers need more familiarity with the online platform, which can affect communication and teaching effectiveness.

**Figure 3:**
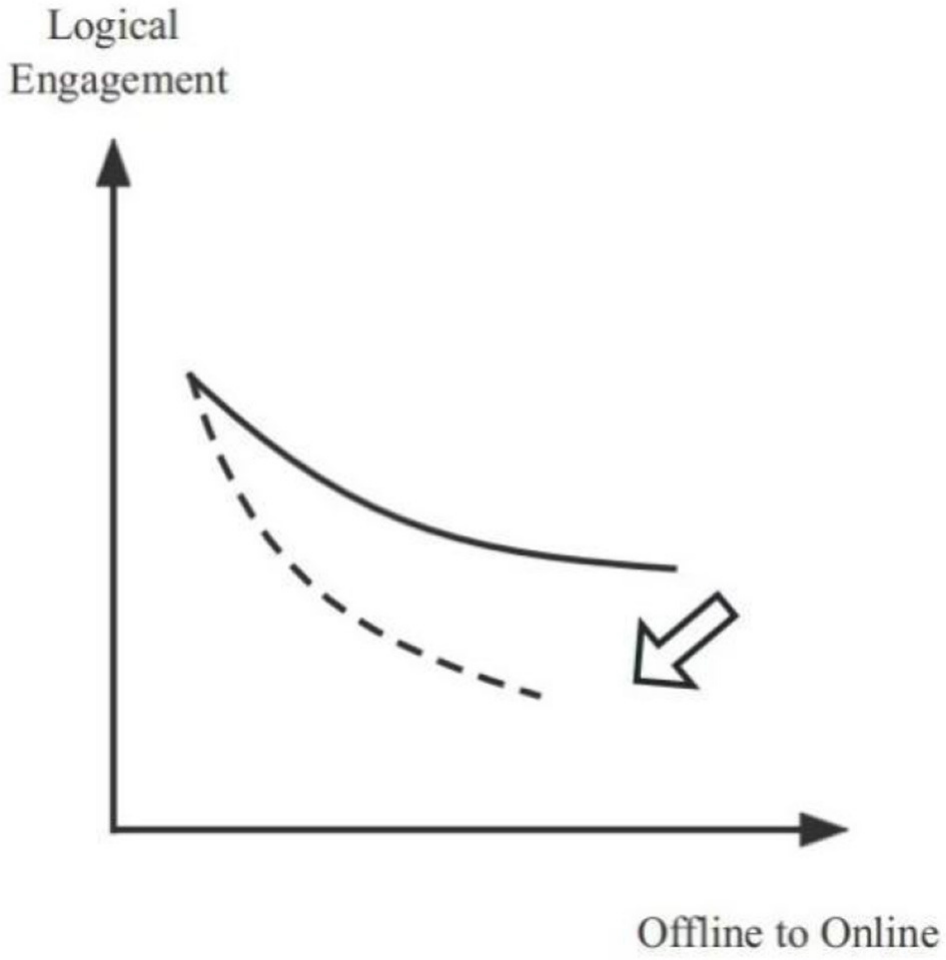
The model of the negative moderating effect of external intervention influences.

Another important external confounding condition is the “learning environment”. *The learning environment is indistinguishable from time and space. I can study at home and also rest at home. The bed was originally used for resting, but sometimes, when I open my eyes, I am in bed for class. Additionally, with the sound of talking and pets barking at home, it is impossible to turn on the camera or open the microphone to communicate*. (F-H-O-230916) Exams and assessments are supposed to be an internal part of the classroom and an element of learning, yet in the online classroom, these have been alienated. One *of my teachers wrote a brief question on an online platform. He told students to keep answering it because he made it a requirement that more than five answers count as attendance*. (M-H-O-230920) Clearly, such a learning environment does not improve efficiency or facilitate communication. The spatiotemporal combination leads to a lack of learning atmosphere: people are likelier to become lazy. The learning method lacks rituals and good change. The alienation of assessment makes the whole process like quality assurance in the “mystery box” (Zhang et al., 2022). All teachers are concerned about is whether the knowledge is delivered in some form and whether students are engaged in learning. However, students may be able to cooperate with such alienated assessments, while others may doubt this kind of assessment lacks fairness and increases learning anxiety. *The online exams are free for everyone to look up information. The teacher will not know if they are plagiarized, so if this is used as a grade, it will have a big impact on me*. (F-S-M-230918) The external intervention condition plays a negative moderating role. From offline to online, the level of logical engagement of students gradually decreases, while the external intervention makes the slope of the negative direction larger (see Figure 3).

The teacher's “pedagogy strategies” are critical internal interventions, including “teaching methods” and “teaching content”. One of the characteristics of online learning is the Human-Computer-Human model, which renders many of the teacher's teaching strategies “unworkable”. Some teachers continue the old offline practice. *If they feel no one is responding to the questions, they will give up and turn off the camera. They will keep reading in a long, continuous voice, and then I want to go to sleep. No, I fell asleep halfway through*. (M-H-O-230715) In contrast to this dull atmosphere, some teachers were clearly more experienced in online teaching. Their teaching is more acceptable, which makes students' engagement in learning significantly higher.

*During offline learning, teachers could arrange classes in dramatic ways or individual performances. In online classes, teachers changed to playing animation videos. They will pause and ask students to discuss what happened in the animation. This format, I think, is exciting and quite appealing to us……* (M-H-O-230715)

*Usually, the online environment is free and popular. If the teacher can refer to the language and format of the internet influencers or program hosts, it will break that dull atmosphere and make it more fun*. (M-H-M-230715)

*If the teacher could use more buzzwords or interesting stickers, you would feel closer to him. Even though the learning content is challenging to understand, you will not be rejectable……* (F-H-M-230918)

*I was handed some colorful textbooks with illustrations when I was learning Portuguese. It will not be boring to read them. Everybody can learn something in this way*. (F-H-M-231018)

**Figure 4:**
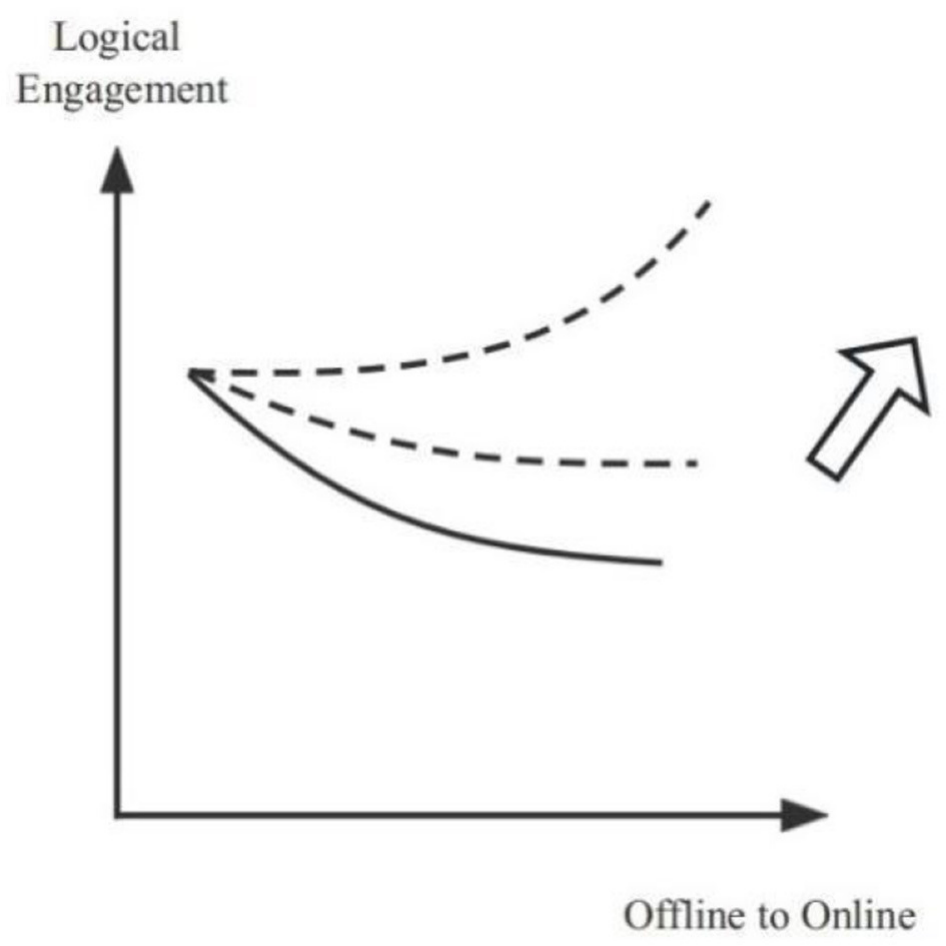
The model of the positive moderating effect of internal intervention influences.

When studying online, students are more sensitive to “teaching content” than offline. Some of the more theoretical content is not popular among students. The effectiveness is even worse if the academic part is combined with the light output teaching method. *This kind of teacher is good at academics, but he or she is not very good at teaching. Their classes are too theoretical, which makes us feel that we are in awe and does not help to activate the classroom atmosphere*. (M-S-O-230718) Online learning often improves engagement if there is unlimited theoretical content combined with constant hands-on practice. *He had many examples so that I could understand the theory better. By practicing again and again in this way, my attention had to be focused because he would ask questions following the practice every few minutes*. (M-SS-M-231018) In comparison, online learning practice is more attractive than theories for students. It is more feasible to deliver practice and even needs to be supported by advanced theory. *After introducing a writing method, there is an immediate hands-on session. You can freely write based on your understanding according to the method. The teacher draws a few students to analyze it on the spot, and the response will be quite good*. (F-H-M-231012) For the intervention of “pedagogy strategies”, when the teaching format is more “acceptable” and the teaching content is more “hands-on”, the students' logical engagement will be increased. Teachers need to be more innovative in how they develop their lesson plans since students love this range of multidimensional and dynamic interaction in practice (Tanis, 2020). The internal intervention makes the otherwise negative slope smaller and may have a positive moderating effect (see Figure 4).

#### 4.3.2 Emotional engagement: relaxed and stress-free learning

The essential feature of online learning is that the learner can learn without joining a real environment. This reduces emotional disturbance and adjustment. Therefore, the entire online learning process is relaxed. Both “self-disciplined” and “undisciplined” students will enjoy this process. Using theoretical sampling in a continuous comparison process, two students at the extremes were selected for interviews. One had won first place in a regional essay competition and was the top student in the class. The other had a poor academic foundation. Both had the same perceptual understanding of online engagement. *I don't have to be so nervous when getting up in the morning. I can boil water at a relatively leisurely pace, put a glass of water in front of the computer, and then sit down again at a leisurely pace. I slowly turn on the computer, feeling the whole process very relaxing and wonderful. I was the only one there, and there was no need for me to put on clothes and shoes to go out and walk exhaustedly, as I do offline*. (F-H-O-230920) *I am a person who gets nervous easily. I think face-to-face learning makes me feel like making a fool of myself. It is much easier for me to study online and take initiative*. (F-SS-M-230715)

Students see “flexibility and independence” as key elements that help them succeed in their online study (Tanis, 2020). Such a feature makes e-learning more stress-free. The biggest difference between face-to-face classes is that there is less learning pressure when studying online. *Knowing that the reports, quizzes, and exams will be taken online, the whole person suddenly becomes relaxed and lazy*. (F-H-M-231018) More self-disciplined students have their own opinions about the lack of pressure. *Unless I have much emotional interaction with this teacher, it seems he does not exist as a real person in my life. This gives me less sense of pressure, and once the learning anxiety becomes less, my autonomy becomes stronger*. (F-H-O-231018) The advantages and disadvantages of online learning can be transformed at any time for different students. For example, stress-free learning may lead to a person becoming lazy, or it may enhance one's autonomy. Whereas external and internal intervention factors have a greater impact on rational input, they have a smaller impact on perceptual input states. Because it originates from a person's prior feelings and perceptions which are not influenced by acquired experiences and intervention factors (Maddy, 2000).

### 4.4 Parallel experiences: “superposition states” of rational dissatisfaction and perceptual satisfaction

The critical factor determining undergraduates' learning experience is their engagement with learning. The duality of engagement also determines the duality of experience. However, it is paradoxical when focusing on the experience. Students' rational and perceptual responses are a “superposition state” of satisfaction and dissatisfaction. Just like a Schrödinger-cat state, an interesting quantum superposition of classically different states may be deterministically produced by using a single atom in a cavity to regulate a propagating optical pulse (Duan, 2019).

Thinking about online learning logically, the Marginal Effect of the teacher's class is significantly lower. Besides, the content of the class is not consistent with the teacher's expectations. *You can clearly feel that teachers teach more things offline compared to online. The teachers also say they cannot expand topics on specific areas and have to skip them*. (M-SS-O-230916) Being in the online human-computer environment, it is difficult for the teacher to have emotional connections with classmates, let alone emotional responses needed for the marginal expansion of knowledge. Therefore, the educational content is correspondingly reduced in depth and breadth. *In online classes, teachers cannot see our eyes, then they may just follow the routine of teaching*. (F-H-O-230715) The root reason for this is that online education's communicative nature is too weak. If teachers could adopt vivid, practical strategies with relatively few external distractions, teachers' knowledge expansion and the marginal benefit to students would be enhanced.

Another rational perspective concerns the difference between learning and learning efficiency. Through the process of coding dimensions, the keyword that can be extracted is “not fully understood”. Students seem to learn the same things as they do offline, but they feel they do not fully understand them. *We are not really sure if we have learned anything, even though we have fairly easy access to the online classroom*. This situation is more due to the lack of efficiency in the online classroom. Most colleges and universities have adopted flexible assessments due to the shift in teaching mode. However, it has resulted in *grades going up, but we learned a little* (M-SS-M-231012). Different student types in the four quadrants responded diversely to the learning situation. Nevertheless, all students expressed their dissatisfaction with the level of knowledge acquisition in the online classroom.

*(Online) learning efficiency is definitely low, but when I know I can learn again through recordings and playback, I follow my own pace and will not care about online problems—*Mind-body integrated type (F-H-O-230920)

*(Online learning) My mood is relaxed. How about the result? You can't mention it... If the teacher knows how to adapt the teaching method, the students will welcome the lessons—*Passive regulated type (M-H-M-230920)

*The best part of online learning is relaxation. The worst part is that I am too relaxed. I cannot be focused in class. It is easy to miss important content*—Emotionally comforted type (F-SS-M-230713)

*It is more tiring than usual if you want to achieve results at the same level as offline because it is impossible to study continuously in online classes. I feel like I am not playing well or learning well—*Self-value added type (F-S-O-230718)

Although almost all students expressed rational dissatisfaction, they simultaneously expressed emotional satisfaction without realizing that they were both satisfied and dissatisfied with learning online. *Online learning is a thing that makes people feel happy, convenient, free, and comfortable. I can learn anytime,... well, but sometimes it does not. It is cold. I cannot adapt because I hate this process*. (F-SS-M-231018) The autonomy and freedom of online learning are perceptual and intuitive experiences. Being unable to adapt or hating is based on rational reflection on knowledge learning. These two seem contradictory, but they can always coexist, just like Schrödinger's Cat's “superposition states”–a parallel experience of the coexistence of sensibility and rationality.

## 5 Discussion—Self-determination theory and parallel experiences

Motivation, interaction, engagement, and pedagogy strategies are interrelated concepts in classroom learning research. Self-determination theory suggests that human motivation is divided into external and natural motivation. Personal decisions are free, active decisions based on capturing information about the external environment and individual needs. Individual needs refer to autonomy, competence and relatedness assumed by Self-determination theory. natural motivation is developed by satisfying these three a priori needs (Deci and Ryan, 2000). College students also have three kinds of psychological needs. Moreover, several regulatory procedures and various goal contents are related to varying levels of need satisfaction (Deci and Ryan, 2000). These needs are solidified in face-to-face learning, where all students have the same physical space. Furthermore, due to the changing characteristics of online learning, the degree of satisfaction of college students' psychological needs has changed. Relatedness and competence are weakened, while autonomy is unrestrictedly enhanced due to spatiotemporal characteristics. Even with internal and external factors moderating and influencing, these critical natural needs only change in degree (slope), but not fundamentally. Many studies on Self-determination theory have shown that autonomy plays a vital role in the internalization of extrinsic motivation (Ruzek et al., 2016; Zhou et al., 2019; Chong et al., 2021); other scholars claimed that Perceived autonomy influences all of the elements of student involvement, but it is not the most important one (Chiu, 2022). The results of this study confirm that autonomy plays a decisive role in college students' perceptual inputs. Personal emotions bring comfort and relaxation experiences, depending on the person's prior feelings. For logical engagement, the role of autonomy depends on the psychological needs of relatedness and competence. Obviously, in online learning, these two psychological needs of undergraduates must be enhanced by external conditions. *If the teacher's class is lively and interesting, it gives us an expectation. Then everyone is willing to participate in the teacher's discussion*. (F-SS-M-230718)

**Figure 5:**
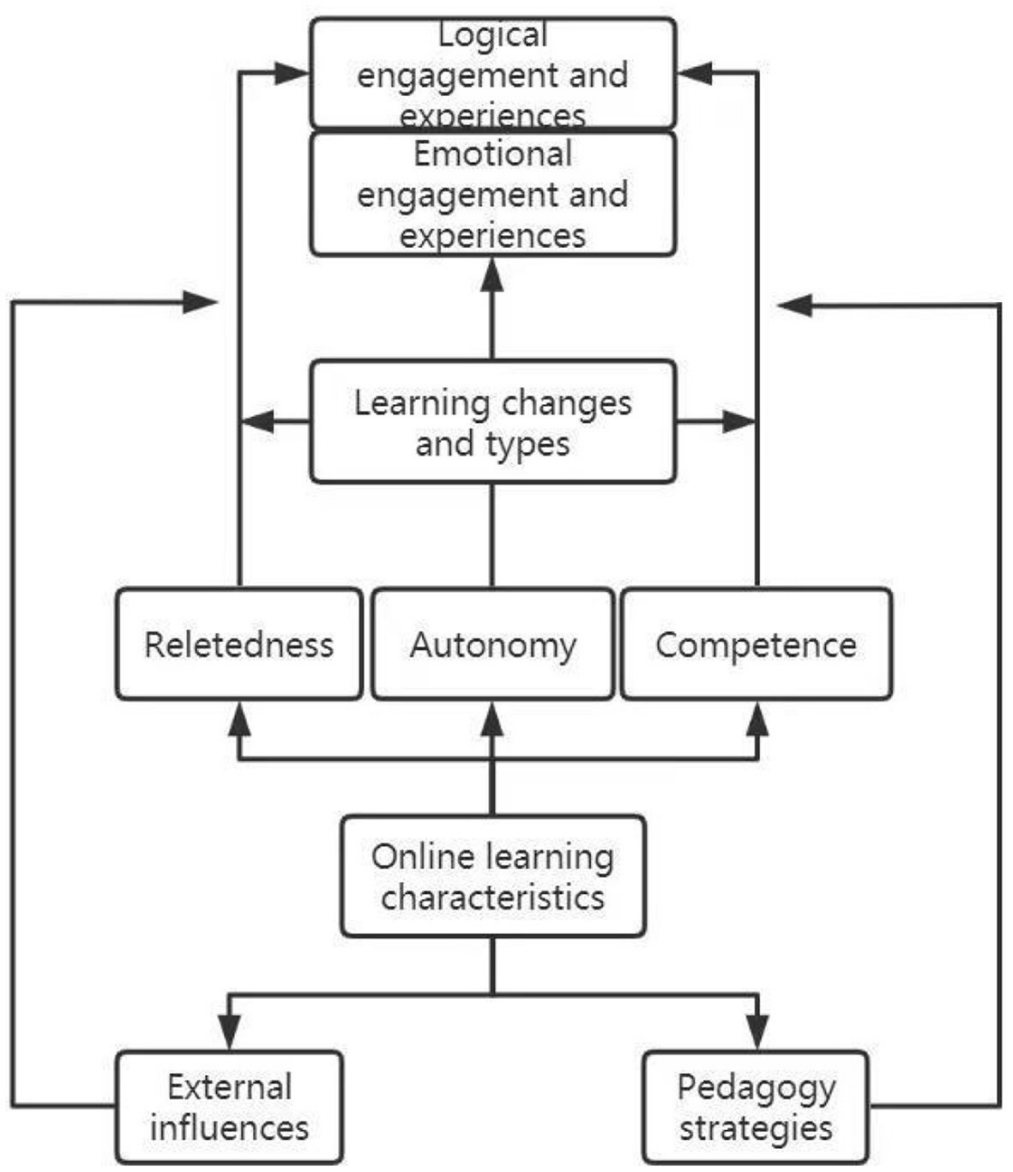
The mechanism of action of the three psychological needs of online learning.

However, as the students mentioned in their interviews, *there is no way to change the poor communication and inefficiency of online teaching*. These two weaknesses in online learning also correspond to a need for more relevance and the psychological need for competence, combined with the involvement of exogenous factors that lead to unsatisfactory rational engagement and experience. Although autonomy is a crucial factor driving perceptual engagement, it also impacts rational engagement and experience. Undergraduates have different perceptions of learning rationality, and the natural motivation brought about by autonomy also differs. A major source of satisfaction and vigor throughout life is natural motivation (Ryan and Deci, 2000). However, through the socialization process of students, autonomy does not necessarily bring enjoyment and vitality to learning. Instead, it is more likely to be enjoyed and tends to be avoided. Students in the first and fourth quadrants undoubtedly have high autonomy and self-discipline in learning. However, the inefficiency of online learning makes them change their learning strategies, either by borrowing the advantages of online learning flexibly and repeatedly or by using external forces to guide the discovery of learning. In conclusion, rational dissatisfaction and perceptual satisfaction are intertwined. The two perceptions are differentiated by the change in the strength of psychological needs and by the two-way penetration of autonomy that makes college students' perceptions appear like quantum trajectories in a superposition state like Schrödinger's Cat. The mechanism of action of the three psychological demands (autonomy, competence and relatedness) in online learning is shown in Figure 5. The result is like the thought experiment conducted by Erwin Schrödinger, “A cat is locked in a closed container with a small amount of radium and cyanide. If the radium decays, the mechanism is triggered, and the cat dies; if the radium does not decay, the cat is alive”. According to Quantum mechanics theory, radioactive radium is in a superposition of decayed and undecayed states. The superposition of microscopic particles should lead to a superposition of macroscopic states, namely, Schrödinger's Cat. However, there can be no cat that is both dead and alive. The conclusion is known only when the container is opened (Trimmer, 1980). Suppose the changes in autonomy, relatedness, and competence in online learning lead to parallel experiences. Then rationality and sensibility are the “container valves” that confirm the satisfaction of the individual student. *If you just talk about the(online) in-class experience, of course, it is relatively more comfortable and relaxing. But if it is about the learning outcome, of course, it is not that satisfying*. (F-S-M-230918)

## 6 Conclusion

This study delves into the online learning experience of undergraduates within the increasingly popular online learning environment. Researchers conceptualize learning contexts, characteristics, motivation, interaction, external influences, and learning engagement as an interconnected whole, exploring their profound influences and connections. Grounded in existing literature, the study employs a qualitative analysis approach grounded in theory, well-suited for identifying pattern features and conducting exploratory analyses. Through constant comparisons and theoretical sampling, a well-developed “storyline” emerges.

In the process of discovering the core category, various elements are elucidated—from antecedent variables to phenomena, situations, external factors, and deeper into inputs and experiences. These elements exhibit potential logical correlations with underlying driving factors, ultimately culminating in the emergence of parallel experiences akin to Schrödinger's Cat. The theoretical connection highlights the significance of basic psychological needs, specifically self-deterministic autonomy, relatedness, and competence. Variations in the satisfaction of these three basic needs among college students in online learning contribute to the emergence of parallel experiences, offering a profound basis for theoretical development and educational enhancement in the realm of online learning.

### 6.1 Theoretical contributions

The Grounded Theory's strength lies in the systematic development of a substantive theory through the identification of core categories within the realm of conceptual coding. The primary theoretical contribution of this study is elucidated in Figure 1, which delineates the logical progression of the storyline and the core category of “Schrödinger's Cat's parallel experience.” It is imperative to clarify that, within a specific learning transition context, the study does not seek to construct a theoretical framework. Rather, it aims to deeply attribute the specificity of online learning experiences, employing the logical path inherent to grounded theory. Despite the constancy of essential elements in the learning process—such as teachers, students, learning objectives, knowledge transfer, teaching methodologies, and assessments—alterations in the external environment can induce changes in students' fundamental psychological needs, thereby giving rise to a parallel experiential phenomenon reminiscent of Schrödinger's Cat.

The second theoretical contribution involves refining the scope of implications within the Self-determination theory. Traditionally perceived as a theory of motivation, the Self-determination theory emphasizes autonomy, relatedness, and competence as psychological needs contributing to internalizing extrinsic motivation and fostering optimal experiences that yield a sense of wellbeing and enjoyable performance. However, existing research predominantly focuses on how behavioral and motivational factors predict students' course selection, with limited attention to the impact of the online learning model on psychological needs and subsequent changes in learning perceptions (McPartlan et al., 2021; Zhou et al., 2021). While previous studies have explored the transformation from external to autonomy, the specificity of the online learning model introduces the integrated type, self-value-added type, and regulated type as statuses and learning styles, as illustrated in Figure 5. These, in turn, drive students' rational engagement and learning experiences. In the context of transitioning from offline to online learning, students' engagement and experience result from the combined influence of self-determination and natural determination. Both self and external factors can regulate satisfaction levels of engagement and experience, albeit with a lesser likelihood of transforming the ultimate parallel experience—a key theoretical implication elucidated by this study.

### 6.2 Educational recommendation

Despite limitations in the online learning experience for undergraduates, enhancing their learning engagement and experience is possible by modifying the moderating effect of external variables.

On the one hand, addressing the weak communication aspects of online learning involves strengthening the development and application of synchronous communication technologies, such as VR, to make interactions more realistic. Technological advancements can significantly regulate students' engagement. In the Human-Computer-Human context, educational institutions should establish effective systems to replicate the in-person learning atmosphere, avoiding alienation of assessment functions, or imposing undue constraints on student learning.

On the other hand, in response to the lack of efficiency in online learning, teachers and students need to improve their digital communication skills for technology-enhanced teaching (Chiu, 2022). Making the Internet a tool for increasing learning efficiency rather than a stumbling block is necessary. In addition, teaching is both a science and an art. A timely shift in pedagogy strategies can often make a significant difference. *If you are learning on the Internet, you have to conform to the characteristics of the Internet. For example, teachers could say some popular slang. It will make students feel they are on the Internet, and their interests will follow..*. (M-H-M-231012) *Such as using Kahoot, BLOOKET's website, and doing games or competitions to let students grab or choose answers. The teacher can, at the same time, zoom to implement a classroom quiz*. (F-SS-O-231018) On the Internet, popularity surpasses steadfastly reading from a book. Similarly, online learning content should be practical to maximize student learning motivation. Even in theoretical courses, practices must closely relate to open the closed physical environment. This approach allows teachers to vividly expand the scope of knowledge, enhancing the marginal effect of online learning and ensuring students learn what they need to.

### 6.3 Limitation and further research direction

The study's primary limitation lies in the sample focus on students from Macau, China, the Mainland, Hong Kong, and Portuguese-speaking countries. While culturally diverse, the findings' generalizability to European and American students is yet to be empirically tested. Considering the influence of different cultures on psychological needs, as emphasized by the self-determination theory (Ryan and Deci, 2020). Future research should explore online learning experiences among students from various cultures to validate this study's findings.

The second limitation is the formulaic nature of the study's dimensional quadrant combinations of motivation and interactivity. These combinations offer a broad categorization of student learning types after a learning change but lack insight into cross-cutting contextual attributes. For instance, the combination of weak interactivity and self-discipline may exhibit characteristics of Mind-body integration along with Self-value added. Future research should focus on specific learning processes, employing detailed and practical classifications with feasible research instruments to portray the characteristics of online student learning vividly and intensively.

In summary, while the human experience is real, the online learning experience resembles a superposition state, distinct from the eigenstate, akin to a quantum phenomenon. Therefore, exploring the feasibility and effectiveness of online learning is crucial, beginning with an examination of the experience. Future studies should uncover the fundamental changes in students' psychological needs that shape the online learning experience, enabling research to better address issues that arise in the teaching and learning process.

## Data availability statement

The raw data supporting the conclusions of this article will be made available by the authors, without undue reservation.

## Ethics statement

The studies involving humans were approved by Pedagogic and Research Affairs Office, Macao Polytechnic University. The studies were conducted in accordance with the local legislation and institutional requirements. The participants provided their written informed consent to participate in this study.

## Author contributions

HZ: Writing – original draft, Writing – review & editing. YL: Writing – original draft, Writing – review & editing. SS: Validation, Resources, Writing – review & editing.
